# Prevalence and Risk Factors of Gestational Diabetes Mellitus in Romania: Maternal and Fetal Outcomes

**DOI:** 10.3390/medicina61020194

**Published:** 2025-01-23

**Authors:** Ait El Haj Iman, Anca Huniadi, Mircea Sandor, Ioana Alexandra Zaha, Ioana Rotar, Cristian Iuhas

**Affiliations:** 1 Faculty of Medicine and Pharmacy, Department of Clinical Disciplines, University of Oradea, 1st December Square 10, 410073 Oradea, Romania; ait.iman@csud.uoradea.ro (A.E.H.I.); msandor@uoradea.ro (M.S.); izaha@uoradea.ro (I.A.Z.); 2 Calla—Infertility Diagnostic and Treatment Center, Constantin A. Rosetti Street, 410103 Oradea, Romania; 3 Pelican Clinical Hospital, Corneliu Coposu Street 2, 410450 Oradea, Romania; 4 Department of Clinical Disciplines, Iuliu Hațieganu University of Medicine and Pharmacy, Strada Victor Babeș 8, 400347 Cluj-Napoca, Romania; iuhascristianioan@yahoo.co.uk

**Keywords:** diabetes, maternal, gestational

## Abstract

*Background and Objectives*: Gestational diabetes mellitus (GDM) is a prevalent condition associated with maternal and fetal complications, including preeclampsia, preterm birth, and neonatal risks. This study investigates the prevalence, risk factors, and socio-demographic and medical determinants of GDM in a Romanian cohort. *Materials and Methods*: This retrospective study analyzed 200 pregnant women aged 22–43, grouped by demographic and health factors. Data included glucose tolerance tests, hypertension, obesity, and socio-demographic evaluations. Statistical analysis, performed in SPSS with *p* < 0.05, used logistic regression to assess variable associations. *Results*: GDM prevalence was 10%, with higher rates in women with obesity (25%, *p* = 0.03) and hypertension (12%, *p* = 0.01). Urban living was significantly linked to obesity and hypertension (*p* = 0.02). Cesarean deliveries occurred in 30% of cases (*p* = 0.02), and term births accounted for 85% (*p* = 0.01). Factor analysis identified two key patterns, with urban-related health risks (obesity and hypertension) and socio-demographic vulnerabilities (marital status and rural residence) increasing GDM risk. *Conclusions*: GDM prevalence underscores the impact of medical and socio-demographic factors, stressing the need for tailored interventions like weight management, glycemic control, and equitable healthcare access to reduce risks and improve outcomes for mothers and infants.

## 1. Introduction

In line with the global epidemic of obesity and its related metabolic disorders, gestational diabetes mellitus (GDM) has emerged as one of the most common complications during pregnancy, posing significant risks for both mothers and their infants [1]. GDM is defined as carbohydrate intolerance or hyperglycemia first diagnosed in the second or third trimester of pregnancy in women without previously known diabetes [2,3]. Its global prevalence ranges from 1% to 28%, varying across regions and populations due to factors such as obesity, hypertension, and lifestyle differences [4]. In Europe, a meta-analysis has reported an average GDM prevalence of 10.9%, highlighting the widespread nature of this condition [5]. Notably, Eastern Europe demonstrates the highest rates, with a prevalence of 31.5%, reflecting a higher burden of risk factors, including obesity, sedentary lifestyles, and possibly genetic predispositions, compared to Northern Europe, where the prevalence is only 8.9% [6]. These regional differences are often attributed to variations in healthcare systems, preventive measures, and lifestyle habits.

Romania is particularly affected by GDM due to its elevated prevalence of risk factors, including obesity and hypertension, which are more common compared to other European countries [7]. Additionally, significant disparities between urban and rural areas exacerbate the challenges in preventing and managing GDM. Urban populations face risks related to unhealthy diets, stress, and sedentary lifestyles, while rural populations experience limited access to healthcare services and economic vulnerabilities [8]. These disparities underline the critical need for region-specific interventions to address these challenges and improve outcomes.

While global research has extensively investigated the prevalence and risk factors associated with GDM, data specific to Eastern Europe, particularly Romania, remain limited. Socio-economic inequalities, healthcare accessibility challenges, and lifestyle differences likely play pivotal roles in shaping GDM prevalence and outcomes in this region [9,10,11]. Despite these realities, there is a notable gap in the literature concerning detailed analyses that consider the unique socio-demographic and medical determinants in Romanian populations. Such research is essential for developing tailored strategies to mitigate risks and improve maternal and fetal outcomes.

The global prevalence of GDM has continued to rise over the past two decades, driven by increasing rates of pre-conception obesity and metabolic disorders among pregnant women [12]. Key risk factors for GDM include advanced maternal age, family history of type 2 diabetes, excessive weight gain during pregnancy, and sedentary behavior [13]. Socio-economic factors, including limited access to healthcare, also contribute to the burden of GDM, emphasizing the importance of monitoring and early intervention to prevent complications [14,15]. These factors are particularly relevant in Romania, where disparities between urban and rural settings exacerbate the burden of GDM.

Untreated gestational diabetes mellitus (GDM) significantly increases the risk of a wide range of maternal and fetal complications, highlighting the critical need for effective management during pregnancy. For mothers, complications may include spontaneous abortion, fetal anomalies, preeclampsia, and an increased risk of cesarean delivery due to complications such as macrosomia. For infants, GDM is associated with neonatal hypoglycemia, hyperbilirubinemia, respiratory distress syndrome, and a higher risk of perinatal asphyxia, as well as increased fetal mortality rates [16]. These risks emphasize the importance of strict glycemic control during pregnancy, which has been shown to significantly improve neonatal outcomes by reducing the incidence of respiratory distress syndrome, birth injuries, and other complications resulting from poorly managed maternal hyperglycemia [17].

In addition to the immediate risks, GDM has substantial long-term implications for both mothers and their offspring. For mothers, it is a known risk factor for the future development of type 2 diabetes, hypertension, obesity, and cardiovascular disease, significantly impacting long-term health outcomes [18,19,20]. Furthermore, the children of mothers with GDM are at heightened risk of obesity, type 2 diabetes, and developmental delays, which can have lasting effects on their health and quality of life [21,22]. These long-term risks underline the critical need for effective, evidence-based management strategies during pregnancy, such as personalized glycemic control plans, regular monitoring, and lifestyle interventions.

Diagnosis of gestational diabetes mellitus (GDM) typically relies on the oral glucose tolerance test (OGTT). This widely used diagnostic tool is most commonly performed during the second trimester of pregnancy. This period is considered critical for identifying GDM, as it coincides with the physiological changes in insulin resistance that occur as pregnancy progresses. The OGTT involves the administration of a 75 g glucose load, followed by measuring blood glucose levels at fasting, one-hour, and two-hour intervals. These thresholds, based on established medical guidelines, are designed to identify deviations from normal glucose metabolism and ensure timely diagnosis and intervention [23,24].

Despite its widespread use, there is no universal consensus on the specific testing criteria for the OGTT, with some countries adopting slightly different protocols based on regional guidelines and healthcare practices [21]. For instance, alternative methods, such as the two-step approach recommended by specific organizations, involve an initial 50 g glucose challenge test followed by a confirmatory 100 g OGTT for positive cases. These variations highlight the ongoing debate over the optimal testing strategy for GDM, underscoring the importance of aligning diagnostic practices with the unique needs of different populations [22,23,24]. By standardizing testing criteria and ensuring timely application, healthcare providers can enhance early detection and management of GDM, thereby reducing the risk of maternal and neonatal complications [23].

Alternative protocols, such as the two-step approach endorsed by the American College of Obstetricians and Gynecologists, involve a 50 g screening test followed by a 100 g OGTT for positive cases [25]. Treatment of GDM prioritizes lifestyle and dietary modifications, regular exercise, and weight management, with medications like metformin or insulin used when necessary [26]. These diagnostic and therapeutic approaches are critical for managing the condition effectively, yet access to these interventions may vary significantly between regions, particularly in low-resource settings like rural Romania.

Although advancements in prediction models and biomarkers, such as proteomic screening, have shown promise in identifying at-risk populations, their complexity and cost limit routine clinical application [27,28,29]. Understanding the determinants and prevalence of GDM is essential for improving prevention strategies and reducing maternal–fetal risks [30]. By focusing on Romania, this study aims to provide detailed insights into the prevalence, risk factors, and socio-demographic disparities associated with GDM, contributing to better-targeted public health interventions.

This study also emphasizes the critical role of early screening and timely intervention in managing GDM. Comprehensive prenatal care, including education on healthy lifestyles and glycemic control, can significantly reduce the prevalence of complications associated with this condition. Public health initiatives tailored to address the specific needs of Romania’s urban and rural populations are essential for mitigating the impact of GDM. These initiatives could include community health programs, increased access to diagnostic tools, and training for healthcare providers to ensure consistent and effective care delivery.

The findings from this research will contribute to a broader understanding of gestational diabetes mellitus (GDM) and its far-reaching implications in Eastern Europe, particularly in regions like Romania where socio-economic disparities and healthcare accessibility challenges significantly influence maternal and neonatal outcomes.

This study aimed to provide a detailed understanding of the impact of GDM and related health conditions on pregnancy and birth outcomes, contributing to improved prenatal care practices and risk management strategies.

## 2. Materials and Methods

This study was a retrospective, observational analysis conducted on a cohort of pregnant women to assess the prevalence and impact of gestational diabetes mellitus (GDM) and other health conditions on maternal and fetal outcomes. The study received approval from the Ethics Committee Center for Diagnosis and Treatment of Infertility “CALLA”, nr.817 on 4 October 2023. Data collection focused on specific demographic, health, and pregnancy-related characteristics.

### 2.1. Study Population

The study enrolled 500 pregnant women, aged 22 to 43 years (mean age: 31 years; median age: 30 years), representing a diverse sample of maternal age during pregnancy. To better understand the impact of gestational diabetes mellitus (GDM) on maternal health, the participants were divided into two distinct groups based on the diagnosis of GDM. The GDM Group consisted of 50 women (10% of the total), all diagnosed with GDM through oral glucose tolerance tests (OGTTs), a standard method for diagnosing this condition. The remaining 450 women (90% of the total) were assigned to the Control Group, as they did not meet the diagnostic criteria for GDM.

In addition to the grouping based on GDM diagnosis, participants were further categorized into three distinct age groups to explore the influence of maternal age on the prevalence of GDM and its associated health complications. A total of 33% of the women were in the 20–30 age range, 50% were between the ages of 31 and 40, and 17% were over the age of 41. This age-based stratification allowed for a comprehensive analysis of how age may contribute to the risk of developing GDM and how age-related factors might affect maternal and fetal health outcomes during pregnancy.

### 2.2. Data Collection and Medical Characteristics

Medical data were obtained from existing medical records to capture conditions such as hypertension at multiple stages of pregnancy. Additionally, participants’ marital status, area of residence, and pregnancy trimester were documented, providing a comprehensive profile that identified socio-demographic factors potentially influencing GDM prevalence and management.

Gestational diabetes mellitus (GDM) was diagnosed based on standard oral glucose tolerance tests (OGTTs) conducted during the second trimester, a critical period for identifying potential cases of GDM. These tests were documented in 35% of the cohort, with statistical analysis showing a significant association between the timing of the evaluation and the diagnosis (*p* = 0.02). The diagnostic thresholds for GDM followed the most recent medical guidelines, ensuring that the diagnostic criteria were consistent with current clinical standards. For individuals identified as being at risk for GDM, blood glucose monitoring protocols were recorded as part of standard care documentation.

The medical records included details of monitoring protocols used to track the progression of blood glucose levels and manage any emerging risks associated with GDM. As documented in patient records, these protocols aimed to minimize potential complications through timely interventions. Information on personalized care plans, including dietary adjustments tailored to the individual’s needs and exercise recommendations suited to each patient’s condition, was also extracted from the records. These interventions were implemented in routine clinical practice to manage GDM and reduce the risk of long-term maternal and fetal health issues.

### 2.3. Statistical Analysis

Statistical analyses were performed using IBM SPSS Statistics Version 28.0 (IBM Corp., Armonk, NY, USA). Descriptive statistics summarized continuous variables as means ± standard deviations and categorical variables as percentages. Comparisons between the GDM and control groups used chi-square tests for categorical data and Mann–Whitney U tests for continuous variables. Logistic regression was employed to calculate odds ratios (ORs) and their 95% confidence intervals (CIs) for key associations, adjusting for potential confounders such as age, BMI, and residence. Statistical significance was defined as *p* < 0.05.

## 3. Results

### Baseline Characteristics

Significant differences were observed for age, obesity, and hypertension between the GDM and control groups (*p* < 0.05), as shown in Table 1.

Table 2 shows maternal birth outcomes, highlighting higher cesarean delivery rates in the GDM group compared to the control group (*p* < 0.05).

The age range in the GDM group was 25 to 41, while in the control group, it was broader, from 22 to 43. In the 20–30-year-old age group, the GDM group had a lower percentage (25%) compared to the control group (37%), which was statistically significant (*p* = 0.03). In the 31–40-year-old age group, the GDM group had a higher percentage (60%) compared to the control group (48%), which was also significant (*p* = 0.02). Participants over 41 years of age were evenly distributed across both groups at 15%.

Obesity was significantly more prevalent in the GDM group (50%) compared to the control group (20%) (*p* = 0.01).

Hypertension was more common in the GDM group (18.8%) than in the control group (10.9%), and the difference was significant (*p* = 0.03).

A slightly higher percentage of participants in the GDM group (62.5%) resided in urban areas than the control group (56%) (*p* = 0.02).

Conversely, more participants in the control group lived in rural areas (44%) compared to the GDM group (37.5%) (*p* = 0.03).

Significant differences between groups, such as obesity, hypertension, and urban environment, suggest that these factors are associated with an increased risk of GDM.

The distribution by age group indicates a higher prevalence of GDM among older women (31–40 years), highlighting the importance of careful monitoring of these patients.

Birth outcomes, as detailed in Table 2, included cesarean section rates of 30% (*p* = 0.02) and vaginal birth rates of 70% (*p* = 0.01). Term births accounted for 85% of cases (*p* = 0.01), while preterm births (<37 weeks) and post-term births (>42 weeks) occurred in 8% (*p* = 0.03) and 7% (*p* = 0.05) of cases, respectively. The distribution of infant sex was nearly balanced, with 48% male births (*p* = 0.03) and 52% female births (*p* = 0.02).

Table 3 presents a detailed comparison of maternal and neonatal outcomes to elucidate further the differences between women diagnosed with GDM and the control group. Significant differences were observed in hypertension, obesity, preterm births, and cesarean delivery rates.

The prevalence of hypertension was significantly higher in the GDM group (18.8%) compared to the control group (10.9%, *p* = 0.03). Similarly, obesity was more frequent among women with GDM (50% vs. 20%, *p* = 0.01). Preterm births occurred in 12% of GDM cases, compared to 7% in the control group (*p* = 0.03), while cesarean deliveries were also more common in the GDM group (40% vs. 28%, *p* = 0.02).

The odds of obesity were significantly higher in the GDM group compared to the control group (OR = 4.0, 95% CI: 2.4–6.7, *p* = 0.01), as were the odds of hypertension (OR = 1.9, 95% CI: 1.1–3.4, *p* = 0.03). Cesarean sections were also more common in the GDM group (OR = 1.7, 95% CI: 1.1–2.7, *p* = 0.02).

Table 4 highlights significant differences in fetal outcomes between the GDM and control groups, emphasizing the impact of gestational diabetes on neonatal health. Newborns from the GDM group had higher average birth weights (3850 ± 450 g vs. 3300 ± 400 g, *p* = 0.02) and lengths (52 ± 2 cm vs. 50 ± 2 cm, *p* = 0.03), with a notably higher prevalence of macrosomia (25% vs. 8%, *p* = 0.01). Additionally, neonatal complications such as hypoglycemia (15% vs. 3%, *p* = 0.01) and shoulder dystocia (5% vs. 1%, *p* = 0.04) were more frequent in the GDM group, alongside a higher incidence of Apgar scores below 7 at 1 min (15% vs. 5%, *p* = 0.03). These findings underscore the need for effective glycemic control during pregnancy to minimize adverse fetal outcomes.

The results indicate significantly higher odds of neonatal complications in the GDM group compared to the control group, including neonatal hypoglycemia (OR = 5.71, 95% CI: 2.21–14.7, *p* = 0.01), macrosomia (OR = 3.83, 95% CI: 1.86–7.92, *p* = 0.01), and shoulder dystocia (OR = 5.21, 95% CI: 1.08–25.16, *p* = 0.04).

Table 5 presents two groups of risk factors identified through analysis. Factor 1, labeled urban-related health risks, demonstrated notable associations with hypertension (0.1520) and obesity (0.1542). Factor 2, titled socio-demographic vulnerability and hypertension, showed the strongest association with hypertension (0.4642) and minor correlations with other variables.

## 4. Discussion

The study provides a detailed insight into the medical and socio-demographic characteristics of the study group, including factors such as age distribution, incidence of hypertension and obesity, marital status, and residential environment, as well as pregnancy complications such as gestational diabetes and cesarean delivery rate.

The distribution by age group shows that the majority of women fall into the 31–40 age range (50%), and studies show that this age group is at increased risk of pregnancy complications, including gestational diabetes.

Hedderson et al. (2010) demonstrated that the risk of gestational diabetes increases significantly in women over 35 years of age who have a metabolic profile more prone to insulin resistance. In the context of our group, this observation is supported by the fact that hypertension has an incidence of 12%, which may aggravate the risk of complications associated with gestational diabetes at older ages [31].

The 25% obesity rate among participants is consistent with global data, which identify obesity as a major predictor of GDM [5]. In our cohort, obesity was more prevalent in urban areas (57.5%), where lifestyle factors such as stress, sedentary behavior, and unhealthy diets may exacerbate this risk. Tailored interventions focusing on weight management, physical activity, and dietary counseling for urban populations are critical to reducing obesity-related pregnancy complications [32].

The prevalence of GDM in this study (10%) aligns with findings from European meta-analyses, which report an average prevalence of approximately 10% (Paulo et al., 2021) [4]. The cesarean delivery rate in GDM patients was 40%, significantly higher than in the control group, reflecting complications such as macrosomia and polyhydramnios [33,34,35]. These findings are consistent with previous studies which identified GDM as a significant risk factor for cesarean delivery [35]. In our cohort, 78% of GDM patients experienced polyhydramnios, which is linked to preterm deliveries, abnormal fetal positions, and premature rupture of membranes, further emphasizing the need for comprehensive monitoring during pregnancy [36,37,38].

The factor analysis revealed two distinct groups of risks. Regarding urban-related health risks, obesity and hypertension were strongly associated with urban environments, where sedentary lifestyles and less healthy diets contribute to insulin resistance and increased GDM risk. These findings align with studies by Jiwani et al. (2012) and Giannakou et al. (2019), which highlight obesity as a leading predictor of GDM globally [3,5,35,39,40,41,42]. Hypertension was associated with rural residence and marital status, indicating that limited access to antenatal care and social support can exacerbate pregnancy complications. Carolan-Olah and Frankowska (2014) emphasized the importance of blood pressure monitoring in rural areas, where antenatal care resources are often scarce [43]. Goyal et al. (2020) further highlighted that women in vulnerable socio-economic situations are at higher risk of poor glycemic control and associated complications [32,40].

Our study highlights a prevalence of GDM of 10%, which is within the average values reported in Europe. However, it makes a valuable contribution by specifically analyzing socio-demographic and medical risk factors in the context of an Eastern European country. In contrast to more developed regions, such as Western Europe or North America, where access to healthcare is more equitable, our results suggest that socio-economic disparities and urban–rural differences significantly influence the risk of GDM [31].

Also, while global studies, such as that by Kim et al. (2021), have identified a higher prevalence of GDM in Asia (15–20%) and among ethnic groups in the USA, our study adds insights into the influence of urban and rural environments in Eastern Europe. Urban regions have a stronger association with obesity and hypertension [1]. This highlights the importance of regional intervention strategies, especially in countries with less developed healthcare infrastructure [1].

In addition, factor analysis’s use to delineate urban and socio-demographic risks offers an original approach rarely found in other regional studies. It may serve as a model for future research in other countries with similar characteristics.

Although the overall sample size is adequate, the smaller number of participants with gestational diabetes (n = 50) may limit the power of statistical analysis for subgroups and the generalizability of conclusions.

For women with a history of GDM, postpartum and inter-pregnancy screenings for glucose abnormalities are critical to identifying and managing risks before conception. Such screenings allow one to address metabolic concerns early, potentially reducing complications in subsequent pregnancies [41,42,43].

Our findings align with global studies that identify obesity as a major predictor of GDM [5]. However, the stronger association observed in urban areas in Romania highlights the need for tailored strategies addressing lifestyle factors and healthcare disparities.

Our findings on the prevalence of GDM (10%) are consistent with studies from neighboring countries such as Hungary (9.1%) and Bulgaria (9.9%), highlighting regional similarities influenced by shared socio-economic and healthcare factors. However, they differ from higher rates reported in Western European countries like Germany (10.0%) and the United Kingdom (4.0%), which may be attributed to differences in diagnostic criteria, population demographics, and access to healthcare services [44]. This comparison contextualizes our results within the broader European landscape and underscores the importance of addressing region-specific risk factors [44].

Our findings on the prevalence of GDM (10%) align with data reported in other European studies. For instance, Simmons et al. highlighted regional differences in GDM prevalence when comparing the IADPSG and WHO criteria, showing comparable outcomes but varying classifications across populations [45]. Similarly, a meta-analysis by Eades et al. reported GDM prevalence rates ranging from 3% to 9% across Europe, with substantial variation due to differences in diagnostic criteria and healthcare access [46].

Comparative studies, such as those by Fadl et al. in Sweden and Todi et al. in India, emphasized how the application of standardized criteria, such as those proposed by the IADPSG or NICE, can significantly impact GDM prevalence rates and associated outcomes [47,48]. These findings underscore the importance of considering regional and diagnostic differences when interpreting GDM data. Future research in Romania should aim to evaluate the applicability of these criteria in the local healthcare setting to optimize early detection and management of GDM.

One limitation of this study is the lack of explicit inclusion of socioeconomic status (SES) and healthcare access as independent variables in the regression models. Future studies should aim to collect detailed SES and healthcare access data to provide a more nuanced understanding of their influence on GDM risk and related outcomes.

Another limitation of this study is the absence of HbA1c measurements, which could provide additional insight into the long-term glycemic control of patients with GDM. Future research will aim to incorporate HbA1c alongside OGTT values for a more comprehensive analysis of glycemic management in these patients.

## 5. Conclusions

Our findings highlight the critical need to improve screening programs for GDM, particularly in rural areas of Romania, where access to prenatal care remains limited. Tailored public health campaigns focusing on the prevention of obesity and hypertension among women of reproductive age could help address key risk factors, while integrating standardized GDM management protocols into the national healthcare system, alongside specialized training for healthcare providers, has the potential to significantly enhance maternal and neonatal outcomes.

Furthermore, these results underscore the importance of addressing medical, social, and environmental factors in managing GDM. Universal screening with an oral glucose tolerance test (OGTT) during pregnancy should be considered mandatory to ensure early detection and management, regardless of individual risk factors. Future research should prioritize expanding the scope of socio-demographic data and evaluating the long-term impacts of targeted prenatal care strategies to reduce maternal and child health disparities in Romania.

## Figures and Tables

**Table 1:** Baseline demographic and clinical characteristics.

Characteristic	GDM Group (n = 50)	Control Group (n = 450)	*p*-Value
Mean Age (years)	32.5 ± 4.1	30.8 ± 5.2	0.04
Obesity (%)	50	20	0.01
Hypertension (%)	18.8	10.9	0.03

**Table 2:** Maternal birth outcomes.

Characteristic	Percentage/Value	*p*-Value
Cesarean Delivery (%)	30	0.02
Vaginal Birth (%)	70	0.01
Preterm Birth (<37 weeks, %)	8	0.03

**Table 3:** Comparison of maternal and neonatal outcomes (GDM vs. Control).

Variable	GDM Group (n = 50)	Control Group (n = 450)	OR (95% CI)	*p*-Value
Hypertension (%)	18.8%	10.9%	1.9 (1.1–3.4)	0.03
Obesity (%)	50%	20%	4.0 (2.4–6.7)	0.01
Preterm Birth (%)	12%	7%	1.8 (1.0–3.3)	0.03
Cesarean Section (%)	40%	28%	1.8 (1.0–3.3)	0.02
Polyhydramnios (%)	78%	N/A—Not Applicable	N/A—Not Applicable	<0.001

**Table 4:** Fetal outcomes and complications in GDM vs. Control Groups.

Parameter	GDM Group	Control Group	OR (95% CI)	*p*-Value
Birth Weight (g)	3850 ± 450	3300 ± 400	3.83 (1.86–7.92)	0.02
Birth Length (cm)	52 ± 2	50 ± 2	5.71 (2.21–14.7)	0.03
Macrosomia (%)	25%	8%	5.21 (1.08–25.16)	0.01
Neonatal Hypoglycemia (%)	15%	3%	3.35 (1.38–8.12)	0.01
Shoulder Dystocia (%)	5%	1%	OR (95% CI)	0.04
Apgar Score < 7 at 1 min (%)	15%	5%	3.83 (1.86–7.92)	0.03

**Table 5:** Risk groups.

Factors	Hypertension	Obesity	Single	Married	Urban	Age
Factor 1 Urban-Related Health Risks	0.1520	0.1542	0.0679	−0.1500	−0.5360	−0.1132
Factor 2 Socio-Demographic Vulnerability and Hypertension	0.4642	−0.0711	−0.0465	−0.1288	0.1584	−0.2046

## Data Availability

The original contributions presented in this study are included in the article. Further inquiries can be directed to the corresponding author.
